# 

**Published:** 1896-03-21

**Authors:** 


**Notices of Births, Deaths, and Marriages** are inserted in this column at a charge of 2s. 6d. for an announcement not exceeding four lines.

**Notice to Advertisers and Subscribers.**

All Advertisements, Orders for Copies of The Hospital and Business Communications should be addressed to **THE MANAGER, (Not to The Editor)**, The Hospital, 428, Strand, London, W.C.

The Cost of Subscription, post free, is as follows:—Per week, 2½d.; per quarter, 2s. 8d.; per half-year, 5s. 3d.; per annum, 10s. 6d. Foreign:—Per Annum, 15s. 2d.; payable, in all cases, in advance.

### NOTICE TO CORRESPONDENTS.

All MS., Letters, Books for Review, and other matters intended for the Editor should be addressed The Editor, The Lodge, Porchester Square, London, W.

The Editor cannot undertake to return rejected MS., even when accompanied by stamped directed envelope.

**"Burdett's Hospitals and Charities."**

Sixth year of publication. 950 pp. Scarlet cloth, gilt lettered. Price 5s. A most complete guide to British, Colonial, Indian, and American Hospitals, Dispensaries, Nursing and Convalescent Institutions, and Asylums. A few complete sets of the preceding five issues of the "Annual" may still be obtained on application to the Publishers, The Scientific Press (Limited), 428, Strand, London, W.C.

**NOTICE.—Vol. XVIII. of "The Hospital" (April to September, 1895), in handsome cloth binding, is on sale at the Office of this Journal. Price 7s. 6d. Cases for binding the Numbers contained in this Volume, 1s. 6d. Index to Vol. XVIII., 2½d., post free. The Monthly Part for March is now ready, price 6d. by post, 8d.**

### The Student of Medicine.

### Vacancies.

The following vacancies are announced this week, the amount of salary in each case being given after the name of the institution:—

### Resident Medical Officers.

Birmingham and Midland Free Hospital for Sick Children.—Resident Medical Officer and Resident Surgical Officer. Salaries £70 and £50 respectively, with board, washing, and attendance. Applications to the Secretary, Children's Hospital, Steelhouse Lane, Birmingham, by April 9th.

Dr. Steevens's Hospital, Dublin.—House Surgeon. Appointment for two years. Salary £100 per annum, with apartments, fire, and light. Applications to the Governors and Guardians of Dr. Steevens's Hospital, Dublin, by March 21st.

Dundee Royal Lunatic Asylum.—Assistant Medical Officer. Salary £100 per annum, with board, lodging, &c. Applications to Dr. Rorie at the Asylum by April 4th.

East London Hospital for Children, Glamis Road, Shadwell, E.—House Surgeon. Board, lodging, &c., provided, but no salary. Applications to the Secretary by April 4th.

Evangelical Protestant Deaconesses' Institution and Training Hospital, Tottenham.—Resident Medical Officer. Salary £60 per annum. Applications to the Director, the Training Hospital, The Green, Tottenham, by March 23rd.

General Hospital, Birmingham.—Two Assistant House Surgeons; must possess surgical qualifications. No salary, but residence, board, and washing provided. Applications to Howard J. Collins, House Governor, by March 28th.

General Infirmary, Leeds.—Resident Surgical Officer. Salary £100 per annum, with board, residence, and washing. Applications to the Secretary of the Faculty by March 20th.

London Homœopathic Hospital, Great Ormond Street, Bloomsbury, W.C. —Resident Medical Officer; legally qualified, and be, or become, a member of the British Homœopathic Society. Appointment for six months. Salary at the rate of £100 per annum, with board and apartments. Applications, with copies of 20 testimonials, to the Secretary-Superintendent by March 31st.

London Homœopathic Hospital, Great Ormond Street, Bloomsbury, W.C. —Assistant Resident Medical Officer; legally qualified, and be, or become, a member of the British Homœopathic Society. Appointment for six months. Salary at the rate of £40 per annum, with board and apartments. Applications, with copies of 20 testimonials, to the Secretary-Superintendent by March 31st.

Oldham Infirmary.—Junior House Surgeon; doubly qualified. Salary £50 per annum, with board and residence. Applications to the Rev. Philip Lancashire, Honorary Secretary, by March 24th.

Victoria Hospital, Folkestone.—House Surgeon. Salary £80 per annum, rising £10 annually to £100, with board, residence, and washing. Applications to the Secretary by March 20th.

West London Hospital, Hammersmith Road, W.—House Physician and House Surgeon. Appointments for six months. Board and lodging provided. Applications to R. J. Gilbert, Secretary-Superintendent, by March 25th.

Weston-super-Mare Hospital.—House Surgeon; married, and doubly qualified. Salary £60 per annum, with board and residence in the hospital. Applications to the Honorary Secretary by March 24th.

West Sussex Asylum.—Medical Superintendent. Salary £450 a year, with unfurnished house (the Committee paying rates and taxes), light, washing, coals, and vegetables. Applications endorsed "Medical Superintendent" to Ernest H. Blaker, Clerk to the Committee, West Pallant, Chichester, Sussex, by March 25th.

### Non-Resident Medical Officers.

Borough of Sunderland.—Medical Officer of Health for the Borough and Port Public Analyst. Salary in all, £525 per annum. Will be debarred from private practice, and be required to devote his whole time to the duties. Must be qualified to practise medicine, surgery, and midwifery. Applications, endorsed "Applications for appointment of Medical Officer of Health and Public Analyst," to F. M. Bowey, Town Clerk, Town Hall, Sunderland, by March 31st.

Brighton Throat and Ear Hospital, 23, Queen's Road, Brighton.—Non-resident House Surgeon. Salary at the rate of £52 per annum. Applications to the Secretary by March 31st.

Charing Cross Hospital.—Assistant Surgeon; must be F.R.C.S.Eng. Applications to Arthur E. Reade, Secretary, by March 25th.

City of London Hospital for Diseases of the Chest, Victoria Park, E.—Assistant Physician; must be F. or M.R.C.P. Lond. Applications to the Secretary by April 9th.

Grosvenor Hospital for Women and Children, Vincent Square, Westminster.—Anæsthetist. Applications to the Secretary by March 25th.

Royal Alexandra Hospital for Sick Children, Dyke Road, Brighton.—Surgeon for the Orthopædic Department; doubly qualified. Applications to be sent under cover to the Chairman of the Medical Committee by March 23rd.

Royal College of Physicians.—Milroy Lecturer. Applications to the Registrar, Royal College of Physicians, Pall Mall East, by April 9th.

University College, Bristol, Faculty of Medicine.—Medical Tutor. Salary £125. Applications to the Dean by March 31st.

Victoria Hospital for Sick Children, Queen's Road, Chelsea, S.W.—Second Honorary Anæsthetist; must be legally qualified. Appointment for one year, subject to annual re-election. Applications to the Secretary by March 21st.

Great Ormond Street, Bloomsbury, W.C.

Secretary-Superintendent.

March, 1896. (1,546)

Demy 8vo., with Two Plates, cloth gilt, over 400pp.,
Price 12s. 6d. net,

### Medical History from the Earliest Times,

By E. T. WITHINGTON, M.A., M.B. Oxon.

London: The Scientific Press (Limited), 428, Strand, W.C.

### FANNIN & COMPANY, DUBLIN.
### PUBLICATIONS.

**A MANUAL FOR MIDWIVES.** By Dr. FLEETWOOD CHURCHILL. Edited by THOS. MORE MADDEN, M.D., F.R.C.S.Ed., Obstetric Physician to Mater Misercordiæ Hospital. Late Master of the National Lying-in Hospital, Dublin.

Crown 8vo. 5th Edition. In the Press.

**CLINICAL SLATE.** Designed by HENRY DAVY, M.B., M.Ch., Univ. Dub., &c. For recording particulars of Private and Hospital Cases. 3s.

**A TEXT-BOOK OF CONTINUED AND ERUPTIVE FEVERS.** By JOHN W. MOORE, B.A., M.D., M.Ch., Univ. Dub., F.R.C.P.I. With Charts and Illustrations. 15s.

**DUBLIN JOURNAL OF MEDICAL SCIENCE.** Edited by JOHN W. MOORE, B.A., M.D., M.Ch., Univ. Dub. F.R.C.P.I., &c. Published monthly—containing Original Communications, Reviews, Abstracts, and Reports in Medicine, Surgery, and Collateral Science. Price 2s. each Number, or 20s. per annum.

### FANNIN AND COMPANY,
41, Grafton Street, Dublin,
MANUFACTURERS of SURGICAL INSTRUMENTS & APPLIANCES.
**Medical Booksellers and Publishers.**
Registered Telegraph Address—"Fannin, Dublin." Telephone No. 198.
Established upwards of Half a Century.

### NOTE CHANGE OF ADDRESS.

TRIONAL. SALOPHEN. SOMATOSE. LYCETOL.

Phenacetine Bayer. Sulphonal Bayer.
Piperazine Bayer.

Wholesale Depôt for all BAYER'S Drug Specialities:
### ELBERFELD FARBENFABRIKEN CO., LTD.,
19, St. Dunstan's Hill, E.C.
*Also at MANCHESTER, GLASGOW, & BRADFORD.*

### SULPHUR-DIOXIDE.
IN SEALED TINS.

FOR ROOM DISINFECTION

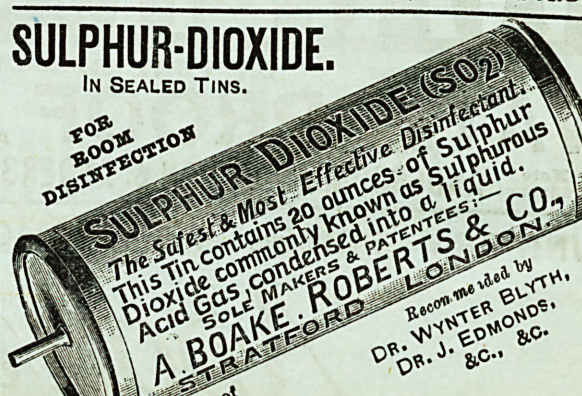

Recommended by DR. WYNTER BLYTH, DR. J. EDMONDS, &C., &C.

To be had of LONDON PATENT MEDICINE DEALERS.
Or of the Makers.

Now Ready, Cloth, 2s. 6d., post free,

### A SKIN PHARMACOPŒIA.

By JAS. STARTIN,
Senior Surgeon to the London Skin Hospital, Fitzroy Square.
Containing concise formulæ for baths, mixtures, ointments, lotions, caustics, rules of diet, classification, and therapeutic index.

Cloth, Bevelled Boards, 2s. 6d., post free,

### EYESIGHT AND SCHOOL LIFE.

**With numerous Illustrations.**
By SIMEON SNELL, F.R.C.S., Ophthalmic Surgeon Sheffield Infirmary.

5s. 6d., post free. Leather, Two strong Pockets, Flap, and Fastener, Gilt Edges, **WRIGHT'S**

### IMPROVED PERPETUAL VISITING LIST.

By ROBERT SIMPSON, L.R.C P. and S.
Requires writing once a month only, and saves much time and labour. Can be begun at any time.
**The Best, Handiest, and most Elegant List issued.**
*Sent on approval to the Medical Profession only.*

**WRIGHT'S NEW IMPROVED**

### CHART HOLDERS,

WITH IMPERISHABLE
***Nickel-Plated Corners and Eyelets.***

Specially made for Hospital use and Private Practice. Any size to order. Much more durable and elegant than those in ordinary use. Prices from 1s. each, in quantities, complete with name of Hospital stamped in gold.

**Samples Free to Hospitals on Application.**

**WRIGHT'S**

### TEMPERATURE AND DIET CHARTS,

For Hospitals, and for Bedside Casetaking.

*Samples Free on application.*

### JOHN WRIGHT and CO., Publishers, BRISTOL.

FOURTEENTH YEAR. NOW READY. 927 pp. 7/6 NET.

### THE MEDICAL ANNUAL, 1896.

**With 20 Full-page Plates, 8 of which are Coloured, and numerous other Illustrations.**

It will be seen that the present issue contains many practical Papers, especially written by Eminent Authorities, and copiously Illustrated in colour and in black and white, forming altogether perhaps the most important Edition that has yet appeared.

*SUMMARY OF CONTENTS AND CONTRIBUTORS.*

**Dictionary of New Remedies. Dictionary of New Treatment. Life Assurance. New Inventions and Improvements in Pharmacy. Books of the Year, &c., &c.**

HERBERT W. ALLINGHAM, F.R.C.S. **Diseases of Rectum.**

FANCOURT BARNES, M.D., F.R.S.E.

E. de la GRANJA, M.D., Boston.

THOMAS MORE-MADDEN, M.D.

PROF. THEOPHILUS PARVIN, M.D., LL.D., Philadelphia.

JOHN W. TAYLOR, F.R.C.S. **Diseases of Women.**

PROF. HENRY DWIGHT CHAPIN, M.A., M.D., New York. **Pediatrics.**

E. HURRY FENWICK, F.R.C.S. **Urinary Surgery.** (*Illustrated.*)

W. SOLTAU FENWICK, M.D., M.R.C.P. **Diseases of the Stomach.**

J. DUNDAS GRANT, M.A., M.D. **Ear Diseases.** (*Illustrated with Two Coloured Plates.*)

F. de HAVILLAND HALL, M.D., F.R.C.P. **Life Assurance.**

PROF. GRÆME M. HAMMOND, A.M., M.D., New York. **Neurology.**

PROF. H. A. HARE, M.D., Philadelphia. **Review of Therapeutics.**

PROF. VAUGHAN HARLEY, M.D., M.R.C.P.

PROF. R. SAUNDBY, M.D., F.R.C.P. **Urinary and Renal Diseases.**

FRANK W. JACKSON, M.D., New York. **Heart Disease.**

OSCAR JENNINGS, M.D., M.R.C.S., Paris. **Remedial Cycling.**

ROBERT JONES, F.R.C.S.

JOHN RIDLON, M.A., M.D., Chicago. **Club Foot.** (*Illustrated with Four Plates.*)

PRIESTLEY LEECH, M.D., F.R.C.S. **Surgery.** (*With Coloured Plate.*)

PROF. BRANSFORD LEWIS, M.D., St. Louis. **Local Anæsthesia.**

WILLIAM MILLIGAN, M.D. **Tuberculous Disease of Middle Ear.** (*With Coloured Plate.*)

NARETH CHANDRA MITRA, M.A., M.B., Bengal. **Indian Diseases.**

PROF. WM. OSLER, M.D., Baltimore. **Typhoid Fever.**

JOSEPH PRIESTLEY, B.A., M.D., D.P.H. **Sanitation.** (*Illustrated.*)

PROF. A. W. MAYO ROBSON, F.R.C.S. **Abdominal Surgery.** (*Illustrated.*)

PROF. GEO. de SCHWEINITZ, M.D., Philadelphia. **Eye Diseases.**

HENRY SEWILL, M.R.C.S., L.D.S. **Diagnosis of Tooth-ache and Neuralgia of Dental Origin.**

W. RAMSAY SMITH, M.B., B.Sc. **Angio-Neurosis.** (*With Two Plates.*)

R. SHINGLETON SMITH, M.D.Lond., B.Sc. **Pulmonary Diseases.**

LANGFORD SYMES, L.R.C.P.I. **Obstinate Hiccough.**

GEORGE THIN, M.D., L.R.C.S. **The Malarial Parasite.** (*With Coloured Plate.*)

WM. THORBURN, M.D., F.R.C.S. **Sensory Distribution of Spinal Nerve Roots.** (*With Coloured Plates.*)

DR. UNNA, Hamburg.

NORMAN WALKER, M.D., F.R.C.P. **Skin Diseases.** (*With Three Plates.*)

P. WATSON WILLIAMS, M.D., M.R.C.S. **Nose and Throat.** (*Illustrated.*)

BRISTOL: JOHN WRIGHT AND CO. LONDON: SIMPKIN, MARSHALL, AND CO., LTD.

Just Published. Third Edition. Re-written and much enlarged. Profusely Illustrated with nearly 50 Plans, Diagrams, &c. Crown 8vo. Cloth gilt. Price 10s. 6d.

### COTTAGE HOSPITALS:

### General, Fever, and Convalescent.

THEIR PROGRESS, MANAGEMENT, AND WORK IN GREAT BRITAIN AND IRELAND, AND THE UNITED STATES OF AMERICA.

WITH AN ALPHABETICAL LIST OF EVERY COTTAGE HOSPITAL AT PRESENT OPENED.

**By HENRY C. BURDETT,**
**Author of "Hospitals and Asylums of the World," "Burdett's Hospitals and Charities," &c., &c.**

The present issue, although a Third Edition, is really a new book on the subject. During the fifteen years which have elapsed since the Second Edition was brought out, Cottage Hospitals have more than doubled in importance and number, so that the Author has had practically to re-write the work. The changes which have taken place, and the opinions of experts, are well brought out, especially in the chapters dealing with construction, where a large number of new plans will be found.

This new Edition is issued in response to the great demand which has existed for the book during the past few years, and the wide interest it has excited amongst many classes of readers, both in this country and in the United States.

"An invaluable book of information on a subject which attracts increasing attention. A complete manual of all that relates to the founding, the cost, and the management of Cottage Hospitals. It embodies the results of wide experience, and it demonstrates the methods by which efficiency and economy may be secured. No more complete manual could be required. It is a pleasure to see the care that has been bestowed on the work, and to bear testimony to its utility. Few half-guineas could be better spent than in its purchase."—*St. James's Gazette.*

LONDON: THE SCIENTIFIC PRESS (LIMITED), 428, STRAND, W.C.

**Hospitals Urgently Needing Annual Subscriptions, Donations, & Legacies.**

### CHARING CROSS HOSPITAL, W.C.

The Hospital is almost entirely unendowed, and relieves over **25,000 PATIENTS YEARLY.** Owing to its CENTRAL POSITION, the Hospital also receives an ABNORMAL number of ACCIDENTS.

**£12,000 in Voluntary Contributions required every year.**

Donations and Annual Subscriptions will be thankfully received by the Bankers, Messrs. DRUMMOND, Messrs. MARTIN and Co., Messrs. COUTTS, Messrs. HOARE, or by the Treasurers.

### NORTH-EASTERN HOSPITAL FOR CHILDREN,

### THE QUARTERLY MEDICAL JOURNAL

FOR YORKSHIRE AND ADJOINING COUNTIES.

Edited for the Promoters by

**SIMEON SNELL, F.R.C.S., Ed., &c.**

Assistant Editors—

D. BURGESS, M.A., M.B.Cantab.<br>W. T. COCKING, M.D.Lond.<br>W. DALE JAMES, M.R.C.S.<br>ARTHUR J. HALL, B.A., M.B.Cantab.

In association with—

A. W. MAYO ROBSON, F.R.C.S., Leeds.<br>P. BOOBBYER, M.B., M.S.Durh., Nottingham.<br>J. A. SOUTHERN, L.R.C.P.Lond., &c., Derby.<br>PRIESTLEY LEECH, M.D., B.S.Lond., F.R.C.S., Halifax.<br>W. L. W. MARSHALL, M.R.C.S., Huddersfield.<br>B. A. WHITELEGGE, B.Sc., M.D.Lond., Wakefield.<br>W. H. JALLAND, F.R.C.S., York.<br>E. H. HOWLETT, F.R.C.S., Hull.<br>ADOLPH BRONNER, M.D., M.R.C.S., Bradford.<br>E. MANSEL SYMPSON, M.A., M.D. Cantab., Lincoln.

**Sheffield: PAWSON and BRAILSFORD.**

Edinburgh and London: YOUNG J. PENTLAND. London: JOHN BALE and SONS.

**Annual Subscription, 6s. 6d. Single Copies, 2s. each.**

### THE BRISTOL MEDICO-CHIRURGICAL JOURNAL.

*Further Permanent Enlargement without increase of price.*

**Published Quarterly, Price 1s. 6d. Annual Subscription, post free, 5s.**

Editor—

**R. SHINGLETON SMITH, M.D., B.Sc., F.R.C.P.,** Physician to the Bristol Royal Infirmary, and Emeritus Professor of Medicine in University College, Bristol. With whom are associated—

J. MICHELL CLARKE, M.A., M.D., M.R.C.P., Physician to the Bristol General Hospital, and Professor of Pathology University College, Bristol.

F. R. CROSS, M.B., F.R.C.S., Ophthalmic Surgeon to the Bristol Royal Infirmary, and Surgeon to the Bristol Eye Hospital.

W. H. HARSANT, F.R.C.S., Surgeon to the Bristol Royal Infirmary.

L. M. GRIFFITHS, M.R.C.S., *Assistant Editor*, Hon. Librarian to the Medical Library, University College, Bristol.

B. M. H. ROGERS, B.A., M.D., B.Ch., *Editorial Secretary*, Physician and Pathologist to the Hospital for Sick Children and Women, Bristol.

Books for Review, Exchange Journals, Articles for Notice in the Section of "Notes on Preparations for the Sick," should be sent to the Assistant Editor, L. M. Griffiths, 9, Gordon Road, Bristol. Subscribers Names and Orders for Advertisements to be sent to B. Rogers, M.D., Editorial Secretary, 11, York Place, Bristol.

**Bristol: J. W. ARROWSMITH. London: J. & A. CHURCHILL.**

Printed by W. H. and L. Collingridge, City Press, 148 and 149, Aldersgate Street, London, E.C.; and Published by the Proprietors, The Scientific Press (Limited), at 428, Strand, London, W.C.

To be had of all Booksellers and Newsagents in the United Kingdom, and at all the Railway Bookstalls of Messrs. W. H Smith and Son.

### THE 3/6 SERIES OF NURSING MANUALS.

**NURSING: ITS THEORY AND PRACTICE.** Being a complete Text-Book of Medical, Surgical, and Monthly Nursing. By PERCY G. LEWIS, M.D., M.R.C.S., L.S.A. Always a standard work on Nursing, it now occupies the position of a classic, being used throughout the world in Training Schools and by Private Students. Eighth Edition. Greatly enlarged and thoroughly revised. Profusely Illustrated. Crown 8vo, cloth, gilt.

"We warmly recommend it as a nursing manual . . . full of useful hints and information."—*Birmingham Medical Review.*

"Full of interest and instruction . . . cannot fail to assist nurses in their work."—*British Medical Journal.*

**DIET IN SICKNESS AND IN HEALTH.** By MRS. ERNEST HART, formerly Student of the Faculty of Medicine of Paris, and of the London School of Medicine for Women. Demy 8vo, blue buckram, gilt, with numerous illustrations.

With an introduction by Sir Henry Thompson, F.R.C.S., M.B. Lond., who says:—"I do not hesitate to express my opinion that the present volume forms a handbook to the subject thus briefly set forth in these few lines, which will not only interest the dietetic student, but offer him, within its modest compass, a more complete epitome thereof than any work which has yet come under my notice."

**OPHTHALMIC NURSING.** By SYDNEY STEPHENSON, M.B., F.R.C.S.E., Ophthalmic Surgeon to the North-Eastern Hospital for Children, Shoreditch, E., &c., &c. This is a valuable contribution to Nursing literature on a subject hitherto never handled with special reference to Nursing. On account of its compact and clear arrangement, and its numerous illustrations, it is specially recommended to the use of the Nursing Profession. Crown 8vo, cloth, 200 pp. Profusely illustrated with Original Drawings, with list of Instruments required, and a Glossary of Terms.

"May very advantageously be studied by nurses and clinical clerks, or dressers, who are about to enter the ophthalmic service of a hospital."—*The Lancet.*

**SURGICAL WARD-WORK AND NURSING.** By ALEXANDER MILES, M.D. (Edin.), C.M., F.R.C.S.E. A practical Manual of Clinical Instruction for Nurses and Students. This book will be found especially useful to beginners, to whom its simple description of elementary principles and treatment in surgery will render it invaluable. Demy 8vo, 200 pp., cloth boards, copiously illustrated.

"Will furnish much-needed guidance to the student and the nurse in the early stages of their apprenticeship."—*Times.*

**ART OF FEEDING THE INVALID.** By a MEDICAL PRACTITIONER and a LADY PROFESSOR OF COOKERY. A popular treatise on the most frequent disorders; with detailed lists of suitable diet and original recipes for daily use. This important work has been undertaken to supply a want long felt by Matrons and the Lady Superintendents in hospitals and institutions of all kinds where invalids are treated, as well as in private households. It is of the greatest value to nurses in private practice. Demy 8vo, cloth gilt, 250 pp.

**OUTLINES OF INSANITY.** A Popular Treatise on the Salient Features of Insanity. By FRANCIS H. WALMSLEY, M.D., Medical Superintendent of the Darenth Asylum, Metropolitan Asylums Board, Member of Council of Medico-Psychological Association. Demy 8vo, cloth extra, 3s. 6d., or popular edition, stitched, 1s. 6d.

A clearly-written manual in which the various features of mental disorder are fully expounded. The author's style is interesting, and the reader's attention is riveted throughout.

"A most useful little book . . . Many of the descriptive chapters in the body of the work are deeply interesting."—*The Medical Magazine.*

London: THE SCIENTIFIC PRESS (Limited), 428, Strand, W.C.

### "THE HOSPITAL"

AN INDEPENDENT JOURNAL OF
**The Medical Sciences**
AND
**Hospital Administration,**
WITH WHICH IS ISSUED WEEKLY
A SPECIAL
**SUPPLEMENT FOR NURSES.**

PUBLISHED EVERY SATURDAY.
PRICE TWOPENCE.

Matrons, Sisters, or Nurses will find in THE HOSPITAL a chronicle of the latest Nursing News, Practical Articles of the highest value to those who wish to keep themselves abreast with the times, and brightly written articles from Nurses all over the world.

SUBSCRIPTIONS CAN COMMENCE AT ANY TIME.

### TERMS OF SUBSCRIPTION.

WEEKLY ISSUE.

| | Great Britain. | Foreign and Colonial. |
|---|---|---|
| For One Year ... ... | 10s. 6d. ... | 15s. 2d. |
| For Six Months ... | 5s. 3d. ... | 7s. 7d. |
| For Three Months... | 2s. 8d. ... | 3s. 10d. |

MONTHLY PARTS.

| | Post Free to any Part of the World. |
|---|---|
| For One Year ... | ... 8s. 6d. |
| For Six Months ... | ... 4s. 3d. |
| For Three Months ... | ... 2s. 2d. |

Postal Orders and Cheques should be made payable to "The Scientific Press, Limited."

N.B.—In order to prevent delay, all business communications should be addressed to "THE MANAGER" only, and not to any person individually.

### CHANGE OF ADDRESS.

In event of change of address, notice should be forwarded to the Publishers by the Saturday previous to date of publication, and the old address should also be given.

"THE HOSPITAL" can be obtained of all Booksellers and Newsagents in the United Kingdom, and at all the Railway Stalls, of Messrs. W. H. Smith and Son; and also from the following Agents in the Colonies and Abroad:—

PARIS: Librairie Galignani, 224, Rue de Rivoli.
BERLIN: Messrs. A. Asher and Co.
LEIPSIG: Mr. F. A. Brockhaus.
NEW YORK: The International News Co.
MONTREAL: The Montreal News Co.
TORONTO: The Toronto News Co.
MELBOURNE, SYDNEY, ADELAIDE, and ZEALAND: Messrs. Gordon and Gotch.
INDIA: At all the Railway Bookstalls and Agencies of Messrs. A. H. Wheeler and Co.
SOUTH AFRICA: Messrs. Juta, Cape Town.

PUBLISHING OFFICES: 428, STRAND, LONDON, W.C

### THE 2/6 SERIES OF NURSING MANUALS.

**HOW TO BECOME A NURSE: AND HOW TO SUCCEED.** A complete guide to the nursing profession for those who wish to become Nurses, and a useful book of Reference for Nurses who have completed their training and seek employment. Compiled by HONNOR MORTEN, Author of "The Nurses' Dictionary," &c. Third and Revised Edition. Demy 8vo, 209 pp., profusely Illustrated with Copyright Portraits and Drawings.

"To those who are frequently appealed to by girls, or young women, as to the steps they should take to become nurses, this book of Miss Morten's must prove a perfect godsend."—*British Medical Journal.*

**THE NURSES' DICTIONARY OF MEDICAL TERMS AND NURSING TREATMENT.** Compiled for the use of Nurses, by HONNOR MORTEN. Containing descriptions of the principal Medical and Nursing Terms and Abbreviations, Instruments, Drugs, Diseases, Accidents, Treatments, Physiological Names, Operations, Foods, Appliances, &c., &c., encountered in the Ward or Sick-room. Third and Revised Edition (25th thousand). Demy 16mo, suitable for the apron pocket, handsomely bound in handsome leather, gilt, price 2s. 6d. net, or in cloth, price 2s.

"Should be at the disposal of every nurse."—*Birmingham Medical Review.*

**HOSPITAL SISTERS AND THEIR DUTIES.** By EVA C. LUCKES, Matron to the London Hospital. The favourable reception accorded to the first two editions of this useful and interesting work, and the regular demand for copies of it, encourage the publishers to place this third edition, greatly improved and enlarged, before the Institutions. The experience and position of its author entitle it to authority, and every Matron, Sister, or Nurse who aspires to become a Sister, should read the advice and information contained in its pages. Third Edition. Enlarged and partly rewritten, crown 8vo, cloth gilt, about 200 pp.

**MENTAL NURSING.** A Text-book specially designed for the instruction of Attendants on the Insane. By WILLIAM HARDING, M.D., M.R.C.P. The want of a complete book for the instruction of Asylum Attendants has been long felt, and it is with confidence that the publishers recommend this work to the managers of all Institutions for the Insane. Second Edition. Enlarged, demy 8vo, profusely illustrated, nearly 200 pp., cloth.

**SPINAL CURVATURE AND AWKWARD DEPORTMENT:** Their Causes and Prevention in Children. By Dr. GEORGE MULLER, Professor of Medicine and Orthopædics, Berlin. English Edition, edited and adapted by RICHARD GREENE, F.R.C.P. Crown 8vo, cloth gilt, with many Plates and Illustrations.

"Dr. Müller's little book is an able essay upon the effect produced by exercise, attitude, and movement upon the growth and development of the body. He further gives directions which any intelligent parent or teacher could carry out with the help of very simple apparatus, showing how to avoid or, in early cases, to cure certain malformations which, in the majority of cases, arise either from the neglect of very simple hygienic rules or from carelessness."—*Daily Chronicle.*

**THE MOTHER'S HELP AND GUIDE TO THE DOMESTIC MANAGEMENT OF CHILDREN.** By P. MURRAY BRAIDWOOD, M.D., formerly Senior Medical Officer to the Wirral Hospital for Sick Children. Crown 8vo, cloth gilt.

"The book is altogether admirable."—*Manchester Courier.*

London: THE SCIENTIFIC PRESS (Limited) 428, STRAND, W.C.

### Midwifery.

*In the Press. Price 5s.*

### BURDETT'S OFFICIAL NURSING DIRECTORY

**A DIRECTORY, NOT A REGISTER.**

**THE HOSPITAL states: "It is well to bear in mind the difference and distinction which exists between a register and a directory. A register implies rights, and suggests that those who are not upon it are in some way or another inferior beings. But a directory is another matter. A directory gives as rights; insertion in it implies no adherence to any particular party or any particular opinion, and in no way interferes with entry on the register of any association, or the list of any institute, to which the individual may belong. All that a directory professes to do is to give information. The 'Medical Register' is not a mere list of qualified medical men; it is admission to the Register, which itself makes him qualified. For years, perhaps even now, 'Churchill's Medical Directory' contained the names of medical men who, so far as public appointments were concerned, were unqualified; it also contains the names of homœopathists, whom four out of every five medical men will not meet; and it certainly contains the names of many for whose moral character we should not like to vouch. But it gives full and accurate information about them all, and for the sake of that information it is constantly consulted both by the public and the doctors."**

WHAT NURSES NEED.

**"At present there is no Register for the nursing profession giving any legal status. Such a thing may come or it may not; but in either case there is no reason why there should not be a Directory, giving such information about the career of each nurse as both the public and the profession want; information, by the by, which no legal Register would be likely to insert. The 'Medical Register' is a bald and meagre thing compared with the 'Medical Directory.' What Burdett's 'Official Nursing Directory' proposes to do is to supply the want of a directory giving information. It will give no rights, it will imply no partisanship, it will give no guarantee except that what it prints is true, and is derived from official sources."**

ACCEPTABLE TO ALL NURSES.

**"This ought to be enough to make it acceptable to the public, and every nurse ought to make sure that, whatever list or register she may be on, her name shall also appear on Burdett's 'Official Directory.'" Applications for forms should be made at once to the Editor, "Burdett's Official Nursing Directory," 428, Strand, W.C.**

**The above work will be ready in the Spring, and those Nurses who have not already sent in their names should at once apply for a form, which can be obtained upon application to the Publishers,**
THE SCIENTIFIC PRESS (Lim.),
428, Strand, London, W.C.

